# *ASPH* Regulates Osteogenic Differentiation and Cellular Senescence of BMSCs

**DOI:** 10.3389/fcell.2020.00872

**Published:** 2020-09-03

**Authors:** Hui Peng, Qi Guo, Ye Xiao, Tian Su, Tie-Jian Jiang, Li-Juan Guo, Min Wang

**Affiliations:** Department of Endocrinology, Endocrinology Research Center, Xiangya Hospital of Central South University, Changsha, China

**Keywords:** *ASPH*, BMSCs, cellular senescence, osteogenesis, aging

## Abstract

Osteogenesis and senescence of BMSCs play great roles in age-related bone loss. However, the causes of these dysfunctions remain unclear. In this study, we identified a differentially expressed *ASPH* gene in middle-aged and elderly aged groups which were obtained from GSE35955. Subsequent analysis in various databases, such as TCGA, GTEx, and CCLE, revealed that *ASPH* had positive correlations with several osteogenic markers. The depletion of mouse *Asph* suppressed the capacity of osteogenic differentiation in bone marrow mesenchymal stem cells (BMSCs). Notably, the expression of *ASPH in vitro* decreased during aging and senescence. The deficiency of *Asph* accelerated cellular senescence in BMSCs. Conversely, the overexpression of *Asph* enhanced the capacity of osteogenic differentiation and inhibited cellular senescence. Mechanistically, *ASPH* regulated Wnt signaling mediated by Gsk3β. Taken together, our data established that *ASPH* was potentially involved in the pathogenesis of age-related bone loss through regulating cellular senescence and osteogenic differentiation, which provides some new insights to treat age-related bone loss.

## Introduction

Osteoporosis is characterized by a reduction of bone mass and dysfunction of bone micro-architecture (Al Anouti et al., 2019; Yang et al., 2019). With the progressive aging of the general population, age-related osteoporosis is becoming a prevalent chronic disease in various countries. Elderly individuals are susceptible to suffer from the age-related osteoporosis because of the loss of bone mass and strength caused by skeletal aging (Li et al., 2015, 2018; Yu et al., 2018; Xiao et al., 2020). Skeletal aging-associated bone loss can be traced back to bone marrow mesenchymal stem cells (BMSCs). BMSCs play a great role in bone formation because of their potential of self-renewal and multi-lineage differentiation including osteogenic lineage (Li et al., 2018; Peng et al., 2019). During aging, BMSCs present with a reduced capacity of self-renewal and osteogenic lineage commitment, which thereby results in the aberrant age-dependent bone formation (Childs et al., 2015; Li H. et al., 2017).

Cellular senescence refers to a state of irreversible cell cycle arrest and distinct cellular alterations in response to various stress (Childs et al., 2015). This process is controlled by various types of tumor suppressors, such as p16^INK4A^, p15^INK4b^, Rb, and p21^CIP1^ (Campisi and d’Adda di Fagagna, 2007; Childs et al., 2015; Li C. et al., 2017). Aging has been reported to be associated with increased cellular senescence. Accumulation of cellular senescence, in turn, can accelerate organismal aging and contribute to the phenotype of aging (Childs et al., 2015). BMSCs will start the aging process early, that is, premature aging, upon stimulated by the stress. Previous studies also revealed that BMSCs underwent cellular senescence during skeletal aging and age-related modulators can cause or prevent premature aging (Lin et al., 2014; Li H. et al., 2017; Zhou et al., 2020).

Aspartate β-hydroxylase encoded by *ASPH* is a type II membrane protein, which includes several domains majorly including N-terminal cytoplasmic domain, transmembrane (TM) domain, Ca^2+^ binding domain and C-terminal catalytic domains (Treves et al., 2000; Finotti et al., 2008). *ASPH* has various isoforms because of its extensive alternative splicing. The longest *ASPH* isoform a containing C-terminal Aspartyl/Asparaginyl beta-hydroxylase catalytic domain can regulate some proteins with the epidermal growth factor (EGF)-like domains (Dinchuk et al., 2000). Based on the current studies, *ASPH* has been reported to be involved in the regulation of tumorigenesis via cell proliferation, colony formation and cellular senescence (Iwagami et al., 2016; Hou et al., 2018). Notably, a Genome-Wide Association Study (GWAS) conducted by Koller et al. (2010) described a SNP located closely with 3’ region of *ASPH.* They found this SNP was conceivably associated with the lumbar spine bone mineral density in the premenopausal European-American women (Koller et al., 2010). In addition, some studies reported that *ASPH* was involved in the regulation of various signaling pathways, such as Wnt signaling, Notch signaling, IGF signaling, IRS signaling and so on (Cantarini et al., 2006; Tomimaru et al., 2013; Iwagami et al., 2016; Hou et al., 2018). It is noted that these signaling pathways are the canonical pathways during bone modeling and remodeling (Malaguarnera and Belfiore, 2014; Tu et al., 2015; Yang et al., 2017). However, the molecular network orchestrating bone formation and cellular senescence of BMSCs mediated by *ASPH* still remain unclear.

In this study, we reported that the level of *ASPH* decreased with aging or senescence. Intriguingly, *ASPH* longest isoform a facilitating the osteogenic differentiation of BMSCs, whereas preventing the process of cellular senescence. Conversely, the knockdown of *ASPH* accelerated the cellular senescence while suppressed the osteogenic differentiation. Mechanistically, these observations might be associated with aberrant Wnt signaling. Taken together, our study revealed *ASPH* promoting the process of osteogenesis while inhibiting cellular senescence through regulating Gsk3β-mediated Wnt signaling, which potentially provided new insights for aged-related bone loss.

## Materials and Methods

### Differentially Expressed mRNAs Filtering and Bioinformatics Analysis

Human mRNA expression data were downloaded from Gene Expression Omnibus (GEO Accession: GEO35955)^1^. The differentially expressed mRNAs between middle-aged and elderly aged groups have been filtered using *t*-test. The R package “limma” (Ritchie et al., 2015) applied to normalize the data and identify DEGs. The DEGs was kept when adjust *p* = 0.01 and | logFC| = 2. Then, kept genes have been assessed the functional enrichments, including GO (Gene Ontology) Biological Processes term and KEGG (Kyoto Encyclopedia of Genes and Genomes) pathway using the R package “Clusterprofiler”(Yu et al., 2012). All probes of the interested *ASPH* gene have been extracted from the raw data. We calculated *ASPH* expression at different coding region according to the recognition sites of probes. Its expressions at different regions then were output the violin figures using *t*-test.

### Co-expression of *ASPH* With Other Genes

The raw expression data of *ASPH* and *RUNX2*, *COL1A1*, *GSK3B*, and *CTNNB1* genes were downloaded from The Cancer Genome Atlas (TCGA) project^2^, the Genotype Tissue Expression (GTEx) program^3^ and CCLE (Cancer Cell Line Encyclopedia) project^4^. The Pearson correlation (r) and *p*-value have been calculated using R and R Studio. The *p*-value has been marked as “0” when it is less than 1^∗^10^–8^.

### BMSCs Culture

Mouse BMSCs (MUBMX-01001; Cyagen Biosciences) were cultured in Mouse Mesenchymal Stem Cell Growth Medium (MUBMX-90011; Cyagen Biosciences). For human BMSCs, we purified from bone marrow of participants underwent hip replacement. The gender of these subjects is male. The ages of young groups were 24, 28, 31, 27, and 32, respectively The ages in old groups were 75, 75, 78, 79, and 85, respectively.

### qRT-PCR Analysis

For analysis of mRNA expression, total RNA from cultured cells was extracted using Trizol reagent (Takara). One-thousand nanograms of RNA was reverse-transcribed into first-strand cDNA using the Reverse Transcription Kit (Takara). For Human BMSCs RNA and cDNA have collected before. qPCR was performed using SYBR Green PCR Master Mix (Takara) and mRNA expression was normalized to reference genes *GAPDH*.

### Western Blot

The protein was lysed using the mixture of RIPA, protease inhibitor (1:100) and phosphatase inhibitor (1:100). Western Blot was performed according to the previous described method (Li et al., 2015). The primary antibodies, GSK-3β Rabbit mAb (#9315; CST), Non-phospho (Active) β-Catenin (Ser33/37/Thr41) Rabbit mAb (#8814;CST), β-Catenin Antibody (#9562;CST), beta Actin mouse monoclonal antibody (#TA811000, ORIGENE) Phospho-GSK-3β (Ser9) Antibody (#9336; CST), and alpha-tubulin (11224-1-AP; Proteintech), were incubated overnight at 4°C, then incubated with appropriate HRP-conjugated secondary antibodies for 1 h at room temperature. The blots were visualized using ECL detection reagents.

### Immunofluorescence

For immunofluorescence, cells were washed by phosphate-buffered saline (PBS) three times. Then, cells were fixed in 4% paraformaldehyde for 15 min and followed by permeabilization and blocking with 7% FCS, 1% TritonX100 in PBS for 30 min at room temperature. Primary antibodies of total β-Catenin (#9562; CST, 1:200) and Non-phospho (Active) β-Catenin (Ser33/37/Thr41) (#8814; CST, 1:800) were incubated overnight in 7% BSA in PBS overnight at 4°C and followed by Alexa Fluor R 488 Goat Anti– rabbit (Thermo Fisher Scientific, United States) at a dilution of 1:400, and finally stained and mounted by mounting medium. Images were visualized on Olympus microscope using cellSense Dimension software.

### Cell Transfection

The *Asph* siRNA, Gsk3b siRNA and the negative control (NC) were purchased from Ribibio (Guangzhou, China). The siRNAs were transfected at the concentration of 100 nM using lipofectamine RNAiMAX (Invitrogen, United States) according to manufacturer’s recommendations. m*Asph* pcDNA3.1-3xFlag-C construct (GenBank:NM_004318.4) was purchased from YouBao Biology. One microgram m*Asph* (AAH) construct and negative control were transfected into BMCSs per well of 6-well-plate using Lipofectamine RNAiMAX (Invitrogen) according to manufacturer’s recommendations.

### Osteogenic Differentiation and Mineralization Assay

To induce osteogenic differentiation of BMSCs, BMSCs were cultured in 6-well plates at 2.5 × 106 cells per well with the mesenchymal stem cell osteogenic differentiation (MUBMX-90021; Cyagen Biosciences). At the 2rd day of osteogenic differentiation, the cell lysates were homogenized for ALP activity assay by spectrophotometric measurement of p-nitrophenol release using an Alkaline Phosphatase Assay Kit (P0321S, Beyotime). At the 7th day of osteogenic differentiation, alkaline phosphatase staining (ALP staining) was performed to evaluate the cell-matrix mineralization. We firstly washed the cells using PBS three times followed by 10% paraformaldehyde for 5 min. Then, cells were incubated in ALP incubation buffer (0.2 g barbital sodium, 0.4 g magnesium sulfate, 0.2 g calcium chloride and 0.3 g beta-glycerophosphate) at 37°C for 2 h. Next, washed cells with 2% calcium chloride and incubated with 2% cobaltous nitrate for 5 min. Then, cells were incubated in 1:80 ammonium sulfate for 10 s. At the 21st day of osteogenic differentiation, Alizarin Red staining was performed to evaluate the cell-matrix mineralization according to the manufacturer’s instructions (MUBMX-90021; Cyagen Biosciences). Briefly, cells were washed using PBS three times followed by 4% paraformaldehyde for 30 min. After washed by PBS for three times, cells were stained in Alizarin red solution at 37°C for 5 min. The stained wells were imaged using the camera (ILCE-5100, SONY). Alizarin Red S released from the cell matrix into the cetyl-pyridinium chloride solution was quantified by spectrophotometry at 540 nm.

### β-Galactosidase Staining

BMSCs with *Asph* siRNA interference were detected by senescence β-Galactosidase staining kit according to the manufacturer’s instructions (G1580, Solarbio Life Science). Briefly, we firstly prepared the staining working solution which comprised of 10 μl β -galactosidase stain A, 10 μl β -galactosidase stain B, 930 μl β -galactosidase stain C and 50 μl X-Gal solution for each well. Then, we rinsed the cell plates with PBS followed by the fixation by 1 ml fix solution of β-galactosidase staining at room temperature for 15 min. After removal of fix solution, the plates were rinsed for three times. Then, cells were incubated with 1 ml staining working solution in incubator at 37°C overnight. Finally, the plates were visualized through Olympus microscope.

### Giemsa Staining

BMSCs with *Asph* siRNA interference was detected by Giemsa staining kit according to the manufacturer’s instructions (G4640, Solarbio Life Science). Briefly, we washed the cell plates with PBS followed by fixation using 1 ml methanol for 2 min. After removal of fix solution, the plates were washed three times using PBS. Then, cells were incubated in 1 ml staining solution for 15 min. Finally, the CFUs were recorded using the camera (ILCE-5100, SONY).

### Statistical Analysis

Data was imported into Excel and scaled and normalized to appropriate controls. Unpaired, two-tailed Student’s *t*-tests were performed for the comparisons of two groups and one-way ANOVA for comparison within multiple groups. Critical *P*-values were Bonferroni corrected and expressed as follows: ns: No significance, ^∗^*P* < 0.05, ^∗∗^*P* < 0.01, ^∗∗^*P* < 0.001, ^###^*P* < 0.001.

## Results

### *ASPH* Is an Age-Dependent Gene in BMSCs

**FIGURE 1:**
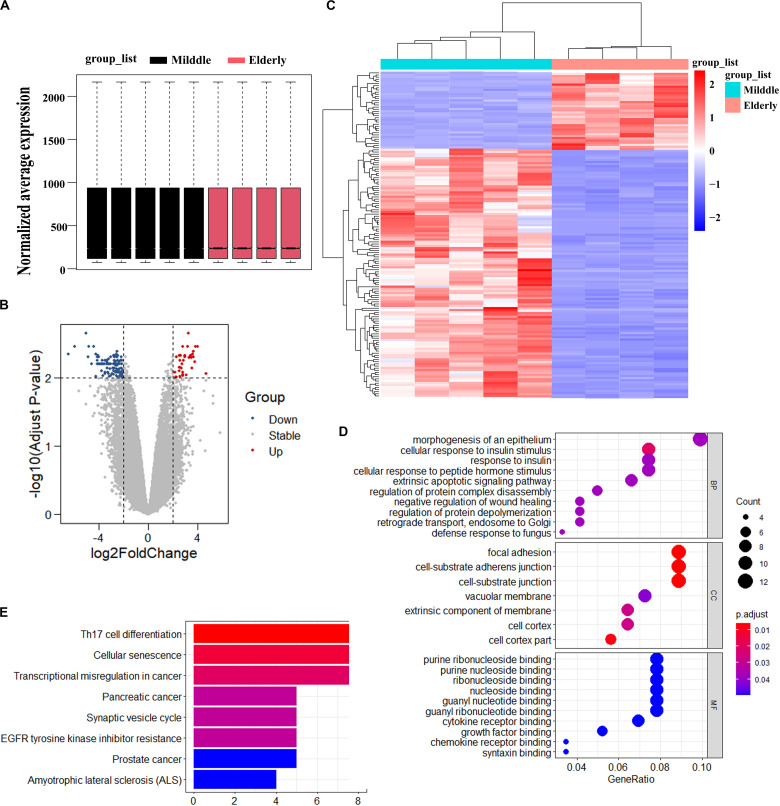
Analysis of differentially expressed genes in BMSCs from middle-aged individuals and elderly aged individuals mined from GSE35955. **(A)** Boxplot of RNA-seq profiling of normalized mRNA expression in BMSCs. **(B)** Volcano Plot of normalized mRNA expression in BMSCs. **(C)** Heatmap of mRNA expression in BMSC. | Fold change| > 2, adjust *p* < 0.01. **(D)** GO (Gene Ontology) analysis of differentially expressed gene in the two groups. **(E)** KEGG (Kyoto Encyclopedia of Genes and Genomes) analysis of differentially expressed gene in the two groups. *p* < 0.05.

To identify the possible causes of age-related bone loss, we analyzed the differentially expressed genes of GSE35955 using R and R studio software (version 3.5) (Raw data was obtained from a public repository of GEO which includes human middle-aged individuals and elderly individuals) (Benisch et al., 2012). Firstly, we normalized the expression of all samples (Figure 1A). Secondly, we kept the deferentially expressed probes through setting the cutoff of adjust *p*-value as 0.01 and LogFC (Log FoldChange) as 2.0, respectively. A total of 152 probes (including 127 genes) have been kept. Of the differentially expressed 152 probes, the expression of 116 probes decreased while the expression of 36 probes increased in elderly aged groups in comparison to the middle-aged group (Figures 1B,C). Thirdly, we analyzed the top 10 enriched signaling using three sub-ontologies (BP for Biological Process, MF for Molecular Function, and CC for Cellular Component) of GO biological processes and KEGG pathway analysis (Figures 1D,E). KEGG analysis showed the differentially expressed genes were majorly enriched in cellular senescence, EGFR tyrosine kinase inhibitor resistance and some cancer development (Figure 1E). Fourthly, we searched potential roles of all genes in bone formation via setting “Senescence OR Aging,” “Bone,” and “Gene symbol (from the differentially expressed genes)” as keywords in Pubmed database. Surprisingly, we found *ASPH* was associated with cellular senescence in cancer and possibly involved in bone health. Thus, we selected “*ASPH*” as our interested gene to conduct further studies.

### *ASPH* Promotes Osteogenic Differentiation

**FIGURE 2:**
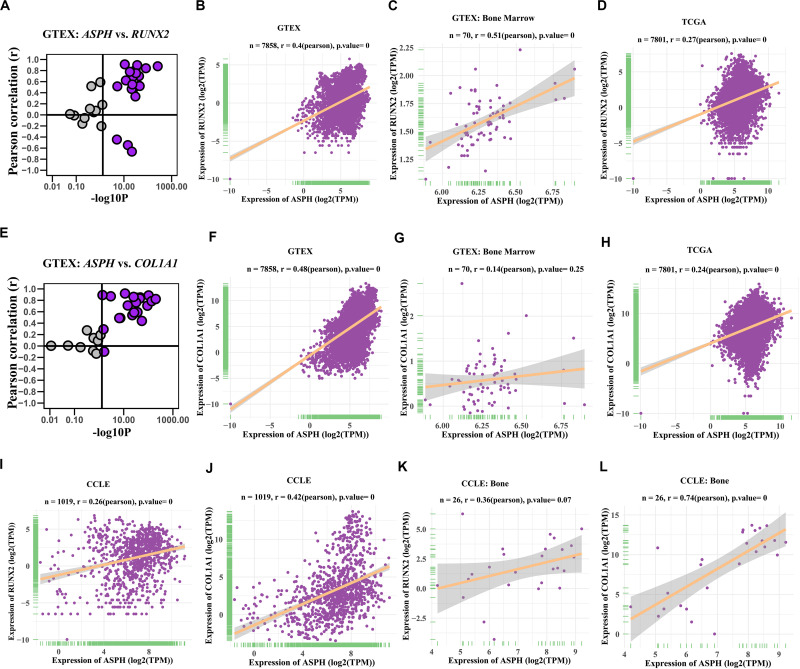
Co-expression of *ASPH* with osteogenic markers. **(A–D)** Correlation of *ASPH* with *RUNX2* in expression in normal tissues **(A,B)**, bone marrow from normal tissues **(C)**, and cancer samples **(D)**, based on the data from Genotype Tissue Expression (GTEx) and The Cancer Genome Atlas (TCGA) databases, respectively. **(E–H)** Correlation of *ASPH* with *COL1A1* in expression in normal tissues **(E,F)**, bone marrow from normal tissues **(G)** and cancer samples **(H)**, based on the data from GTEx and databases, respectively. **(I,J)** Correlation of *ASPH* with *RUNX2* in expression in cancer cell lines **(I)** and in bone related cancer cell lines **(J)**, based on the data from Cancer Cell Line Encyclopedia (CCLE). **(K,L)** Correlation of *ASPH* with *COL1A1* in expression in cancer cell lines **(K)** and in bone related cancer cell lines **(L)**, based on the data from CCLE. The correlation coefficient (r) and *P*-value were calculated by Pearson’s Correlation analysis. Note that every dot represents one tissue type **(A–C,E–G)** or one cancer type **(D,H)** or one cell line **(I–L)**.

Previous studies indicated *ASPH* might be associated with bone mineral density (Koller et al., 2010; Mantila Roosa et al., 2011; Benisch et al., 2012). To investigate if *ASPH* plays a role in bone formation, we analyzed the co-expression of *ASPH* with osteogenic markers such as *RUNX2* and *COL1A1* in various databases. In most of normal tissues from GTEX, the correlations of *ASPH* with *RUNX2* and *COL1A1* were positive, which presented in the upper right region of Figures 2A,E. *ASPH* had a moderate positive correlation with *RUNX2* both in 7858 tissues (Pearson *r* = 0.4, *p* = 0) (Figure 2B) and specifically in 70 tissues of bone marrow (Pearson *r* = 0.51, *p* = 0) (Figure 2C). Similarly, *ASPH* had a positive correlation with *COL1A1* both in 7858 tissues (Pearson *r* = 0.48, *p* = 0) (Figure 2F) even though didn’t show significant correlation with *RUNX2* in bone marrow (Figure 2G). In 7801 tumor tissues, *ASPH* also showed a positive correlation with *RUNX2* as well as *COL1A1* (Figures 2D,H). Moreover, in 1019 cancer cell lines, there were positive correlation between *ASPH* and *RUNX2* (Figure 2I) or *COL1A1* (Figure 2J). Notably, the 26 bone-related cell lines did not show significant co-expression between *ASPH* and *RUNX2* (Figure 2K), but showed positive correlation between *ASPH* and *COL1A1* (Pearson *r* = 0.74, *p* = 0) (Figure 2L). Collectively, these results partially supported that *ASPH* was positively correlated with these two genes (Figures 2A–L).

**FIGURE 3:**
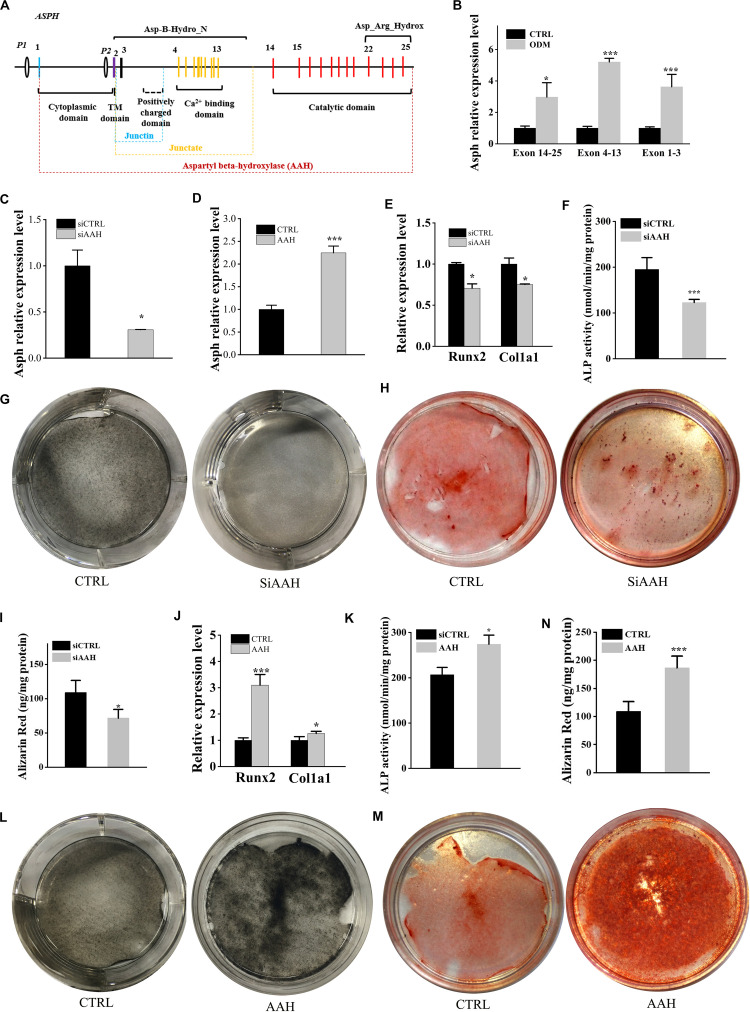
*ASPH* promotes osteogenic differentiation. **(A)** schematic graph for *ASPH* isoforms. **(B)** qRT-PCR analysis of *ASPH* regions’ expression during osteogenic differentiation. **(C)** qRT-PCR analysis of depletion of AAH. **(D)** qRT-PCR analysis of overexpression of AAH. **(E)** qRT-PCR analysis of the relative levels of *Runx2* and *Col1a1* in BMSCs with depletion of AAH. **(F)** Analysis of ALP activity in BMSCs with depletion of AAH. **(G,H)** Representative images of ALkaline Phosphatase (ALP) staining **(G)** and Alizarin red S staining **(H,I)** in BMSCs with depletion of AAH. **(J)** qRT-PCR analysis of the relative levels of *Runx2* and *Col1a1* in BMSCs with overexpression of AAH. **(K)** Analysis of ALP activity in BMSCs with overexpression of AAH. **(L,M)** Representative images of ALP staining **(L)** and Alizarin Red S staining **(M,N)** in BMSCs with overexpression of AAH. These experiments were replicated three times. Error bars show standard deviation. *P < 0.05, ***P < 0.001.

*ASPH* is an 85KD type II transmembrane protein which can generate various splicing variants including aspartyl beta-hydroxylase (AAH), junctin, and junctate (Figure 3A). The longest isoform a (GenBank:NM_004318.4) is comprised of several domains, majorly including N-terminal cytoplasmic domain, transmembrane (TM) domain, Ca^2+^ binding domain and C-terminal catalytic domains. We extracted the expression of all “*ASPH*” probes and classified them into three types according to the probe-targeting positions (e.g., Exon 14–25 with C-terminal catalytic domain, Exon 4–13 with Ca^2+^ binding domain, and Exon 1–3 with a positively charged domain) (Figure 3A). Next, BMSCs were induced by osteogenic differentiation medium and tested its expression of different domains of *ASPH*. The qRT-PCR data showed increased expression of mouse *Asph* during osteogenesis of BMSCs (Figure 3B). Subsequently, BMSCs were transfected with mouse *Asph* siRNA targeting the catalytic domain (Figure 3C) followed by induction by osteogenic differentiation medium. qRT-PCR data suggested that downregulation of *Asph* expression was accompanied by a decline of osteogenic markers at the 7th day, including *Runx2* and *Col1a1* (Figure 3E). Furthermore, the matrix memorization measured by Alkaline phosphatase (ALP) activity, ALP staining and Alizarin Red staining showed a decline of AAH expression resulted in the impairment of osteogenesis in BMSCs (Figures 3F–I). Conversely, BMSCs were transfected with the construct of longest mouse *Asph* isoform variant 1 (GenBank:NM_023066.3) (Figure 3D) followed by induction by osteogenic differentiation medium. qRT-PCR suggested that up-regulation *Asph* expression was correlated with the increase of osteogenic markers, *Runx2* and *Col1a1* (Figure 3J). Similarly, matrix memorization measured by ALP activity, ALP staining and Alizarin Red staining showed over-expression of *Asph* resulted in the enhancement of osteogenesis in BMSCs (Figures 3K–N).

### Ablation of *ASPH* Results in Premature Senescence of BMSCs

**FIGURE 4:**
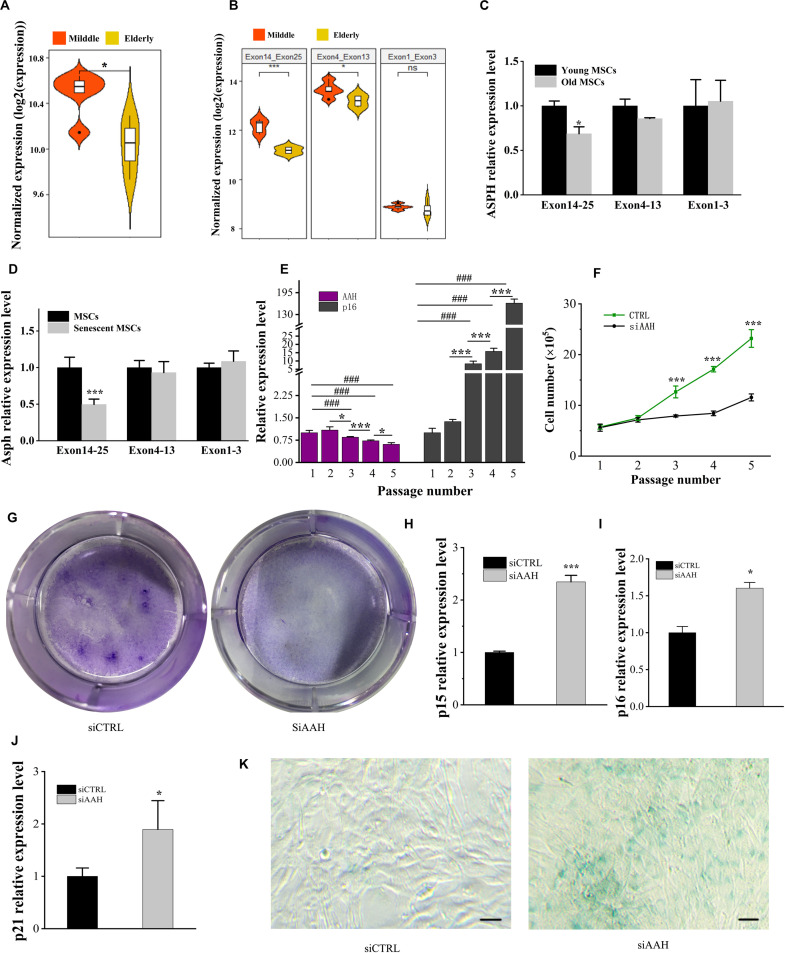
Deficiency of *ASPH* accelerates cellular senescence in BMSCs. **(A)** Mean expression of *ASPH* in the BMSCs from middle-aged individuals and elderly aged individuals (Raw data was obtained from GSE35955). **(B)** Mean expression of different *ASPH* regions in the BMSCs from middle-aged individuals and elderly aged individuals (Raw data was obtained from GSE35955). **(C)** The expression of *ASPH* isoforms in the BMSCs from young and old individuals. **(D)** The expression of *Asph* isoforms in the young or senescent BMSCs. **(E)** The negative correlation of AAH and p16 expression during passaging (P1–P5) of BMSCs. **(F)** Doublings of BMSCs interfered with AAH siRNA *in vitro*. **(G)** Giemsa staining for the CFU-F colonies of BMSCs from BMSCs interfered with AAH siRNA *in vitro*. **(H–J)** Expression of p15 **(H)**, p16 **(I)**, and p21 **(J)** in BMSCs with depletion of AAH. **(K)** Representative images of SA-β-Gal staining of BMSCs in *Asph* siRNA transfected and control group. Scale Bar = 100 μm. These experiments were replicated three times. Error bars show standard deviation. *P < 0.05, ***P < 0.001, ^###^*P* < 0.001.

*ASPH* is known to be involved in the cellular senescence in hepatocellular carcinoma and gliomas (Iwagami et al., 2016; Sturla et al., 2016). In order to understand if *ASPH* is a cellular senescence-related gene in BMSCs, we calculated the mean *ASPH* expression and expression of different ASPH region (Supplementary Table 1; Peng et al., 2020a). The result suggested *ASPH* was lower in elderly aged individuals than middle-aged individuals (Figure 4A). The exon 4–13 and exon 14–25 of *ASPH* both decreased with aging in the two groups. However, the exon 1–3 expression locating at the common region of different *ASPH* isoforms was stable (Figure 4B). Then, we collected human BMSCs (hBMSCs) with different ages and tested their *ASPH* expression at different regions. As expected, compared to the aged group (age from 75 to 90), the young group (age from 24 to 32) showed a higher expression of *ASPH* longer isoforms (Figure 4C). Additionally, we tested the *Asph* expression in young mouse BMSCs (passage 0) and senescent mouse BMSCs (passage 5). Consistently, the expression of *Asph* longer isoforms were lower in senescent BMSCs than in young BMSCs (Figure 4D). These observations suggested that senescence suppressed *Asph* expression. To further test this hypothesis, we performed a series of functional analysis. qRT-PCR results demonstrated that the *Asph* expression was remarkably decreased along with cellular senescence (Figure 4E). Conversely, the senescence-associated p16 expression was positively correlated with cellular senescence (Figure 4E). Additionally, an impairment of the replicative capacity was observed in *Asph* siRNA transfected group (Figure 4F). CFU colonies obtained from Giemsa staining further showed the inhibition of cell proliferation in BMSCs interfered with *Asph* siRNA (Figure 4G). qRT-PCR results revealed that the depletion of *Asph* promoted markers of senescence, such as p15, p16, and p21 expression in BMSC (Figures 4H–J). Consistently, the senescence-associated β-galactosidase staining revealed that the deficiency of *Asph* promoted the cellular senescence (Figure 4K).

### *ASPH* Regulates Wnt Signal Mediated by Gsk3β

**FIGURE 5:**
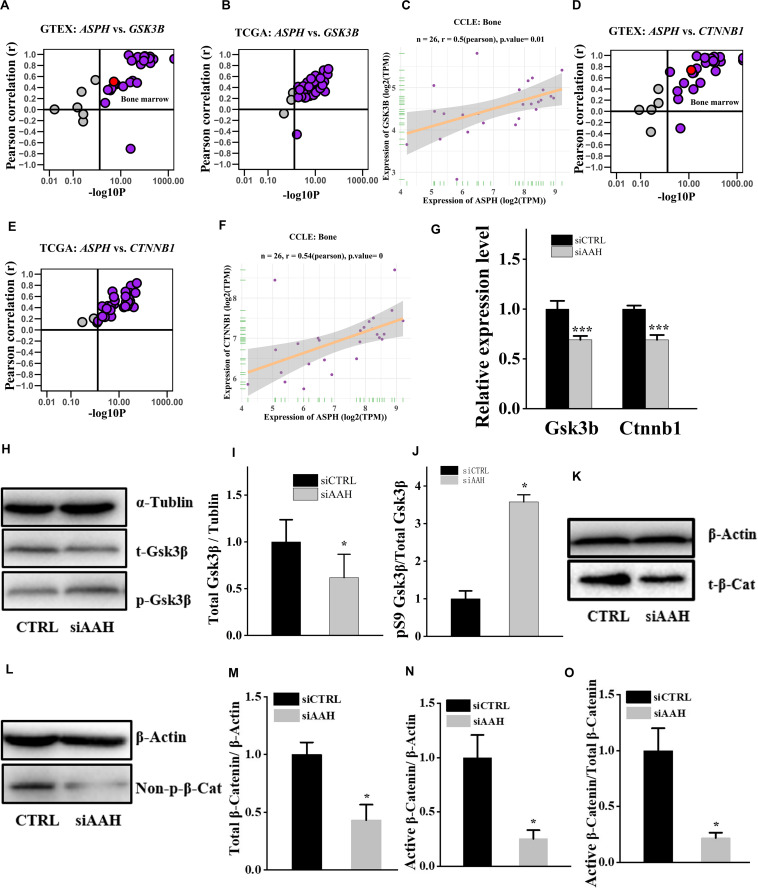
*ASPH* regulates Gsk3β and β-Catenin. **(A–C)** Co-expression of *ASPH* with *GSK3B* in expression in normal tissues **(A)**, cancer samples **(B)**, and bone-related cell lines **(C)**, based on the data from GTEx, TCGA, and CCLE databases, respectively. **(D–F)** Co-expression of *ASPH* with *CTNNB1* in expression in normal tissues **(D)**, cancer samples **(E)**, and bone-related cell lines **(F)**, based on the data from GTEx, TCGA, and CCLE databases, respectively. The correlation coefficient (r) and *P*-value were calculated by Pearson’s Correlation analysis. Note that every dot represents one tissue type **(A,D)** or one cancer type **(B,E)**. The red dots indicate tissue of bone marrow. **(G)** qRT-PCR analysis of *GSK3B* and *CTNNB1* in BMSCs interfered with *Asph* siRNA *in vitro*. **(H)** Western Blot analysis of the levels of total GSK3β and phos-Ser9 GSK3β in BMSCs transfected with *Asph* siRNA. **(I)** The ratio of total Gsk3β to α-Tublin in BMSCs transfected with *Asph* siRNA. **(J)** The ratio of phos-Ser9 GSK3β (pS9 GSK3β) to total GSK3β in BMSCs transfected with *Asph* siRNA. **(K)** Western Blot analysis of the levels of total β-Catenin (t-β-Cat) in BMSCs transfected with *Asph* siRNA. **(L)** Western Blot analysis of the levels of active β-Catenin [Non-phospho β-Catenin (Ser33/37/Thr41): Non-p-β-Cat] in BMSCs transfected with *Asph* siRNA. **(M)** The ratio of total β-Catenin to β-Actin in BMSCs transfected with *Asph* siRNA. **(N)** The ratio of active β-Catenin to β-Actin in BMSCs transfected with *Asph* siRNA. **(O)** The ratio of active β-Catenin to total β-Catenin in BMSCs transfected with *Asph* siRNA. These experiments were replicated 3 times. Error bars show standard deviation. *P < 0.05, ***P < 0.001.

**FIGURE 6:**
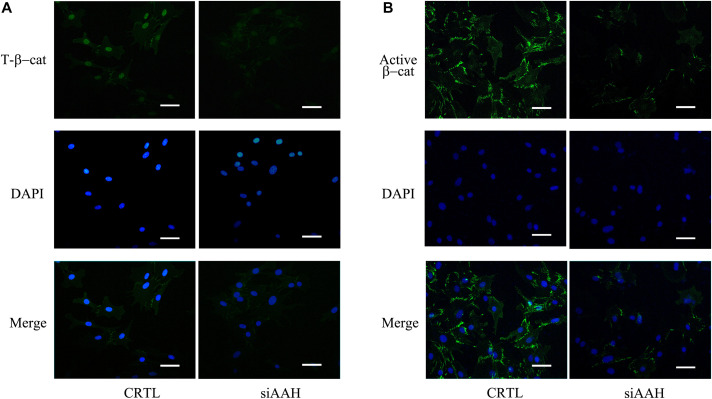
Subcellular localization of total and active β-Catenin. **(A)** Subcellular localization of total β-Catenin protein in BMSCs transfected with *Asph* siRNA. Nuclei, DAPI (blue), total β-Catenin (green). **(B)** Subcellular localization of active β-Catenin [Non-phosph-β-Catenin (Ser33/37/Thr41)] protein in BMSCs transfected with *Asph* siRNA. Nuclei, DAPI (blue), active β-Catenin (green). Scale bar, 250 μm.

**FIGURE 7:**
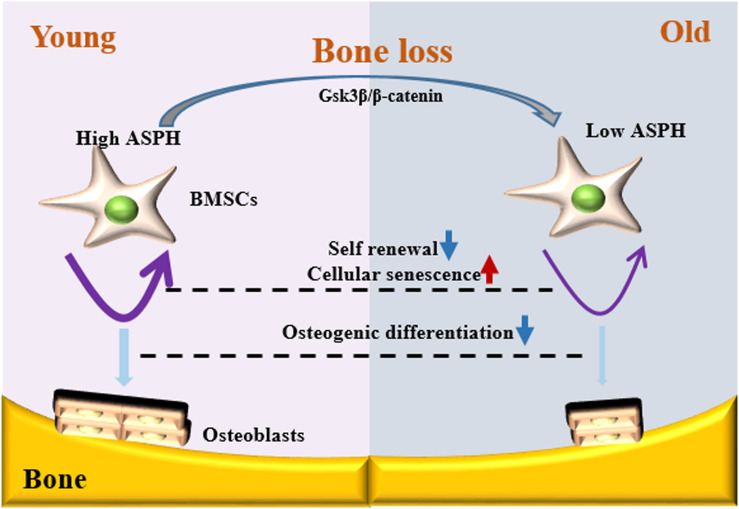
Schematic representation of *ASPH* regulating cellular senescence and osteogenic differentiation of BMSCs during aging. Low expression of *ASPH* restrains the accumulation and activation of Gsk3β and β-Catenin to maintain the capacity of self-renewal and osteogenic differentiation. In the old individuals, the decrease of *ASPH* is companied with the cellular senescence and impaired osteogenic differentiation of BMSCs.

It is well established that Gsk3β and β-catenin have important roles in bone formation. Based on the involvement of *ASPH* in Wnt signaling and cellular senescence in various cancers, we hypothesized that *ASPH* was involved in the bone formation and cellular senescence through regulating Gsk3β and β-catenin (Iwagami et al., 2016; Peng et al., 2020b). Thus, we analyzed the co-expression of *ASPH* with *GSK3B* and *CTNNB1* in GTEX, TACC and CCLE databases. Intriguingly, the analysis showed that *ASPH* has considerable positive correlations in mRNA expression with *GSK3B* and *CTNNB1* in most of normal/tumor tissues (Figures 5A,B,D,E). Furthermore, *ASPH* has a moderate or strong correlation with *GSK3B* and *CTNNB1* in the normal tissues including bone marrow from GTEX database (Tables 1, 2). In bone-related cancer cell lines, *ASPH* has significant correlations with *GSK3B* (Pearson *r* = 0.50, *p* = 0.01) (Figure 5C) and *CTNNB1* (Pearson *r* = 0.54, *p* = 0) (Figure 5F). Consistent with these correlations, the expression of *GSK3B* and *CTNNB1* showed significant decrease in BMSCs with depletion of *Asph* (Figures 5G–O). The ratio of phos-Ser9 GSK3β to total Gsk3β has increased in the *Asph* siRNA transfected BMSCs (Figure 5J). *Asph* inhibition lead to the downregulation of total β-Catenin as well as active β-Catenin (Figures 5K–O). Consistently, cells transfected with *Asph* siRNA showed predominantly decreased distribution of the β-Catenin protein both in cytoplasm and nucleus (Figure 6A). The immunofluorescence analysis also suggested *Asph* inhibition ameliorated distribution of active β-Catenin in cytoplasm (Figure 6B). Taken together, these results suggested that *Asph* possibly regulated Gsk3β/β-catenin signaling, contributing to bone formation and preventing cellular senescence (Figure 7).

**TABLE 1:** The co-expression of *ASPH* with *GSK3B*.

	Total samples	Pearson correlation(r)	*p*-value
GTEX	7858	0.59	0
GTEX: Bone marrow	70	0.51	0
TCGA	7801	0.39	0

**TABLE 2:** The co-expression of *ASPH* with *CTNNB1*.

	Total samples	Pearson correlation(r)	*p*-value
GTEX	7858	0.77	0
GTEX: Bone marrow	70	0.74	0
TCGA	7801	0.48	0

## Discussion

Osteogenesis and cellular senescence of BMSCs play great roles in bone formation (Qadir et al., 2020). In this study, our data showed *ASPH* longest isoform promoting the osteogenesis while inhibiting cellular senescence, indicating it potentially results in an elevated capacity of bone formation. Mechanistically, *ASPH* modulated the accumulation and activation of Gsk3β and β-catenin. In general, our study revealed *ASPH* was an aged-dependent modulator to regulate osteogenic differentiation and senescence in BMSCs (Figure 6).

Aspartate β-hydroxylase (*ASPH*) is a type II membrane protein, which is comprised of several domains, such as N-terminal cytoplasmic domain, transmembrane domain, and C-terminal catalytic domain (Finotti et al., 2008). Through data mining in GEO database, we found some genes differentially expressed in middle-aged subjects in comparison with elderly aged subjects. Through a set of filtering criteria as described in “Results” section, we finally hypothesized *ASPH* gene was partially involved in bone formation. *ASPH* is associated with the regulation of protein complex disassembly and regulation of protein depolymerization (Lee et al., 2012), which has been presented in the top 10 signaling through Go analysis (Figure 1D). Furthermore, KEGG analysis showed that the differentially expressed genes enriched in cellular senescence, EGFR tyrosine kinase inhibitor resistance and some cancer development. These observations gave us some clues that *ASPH* might be involved in bone modeling through regulating cellular senescence.

Accumulated evidence demonstrated that *Asph* was highly expressed during embryogenesis, which is crucial to maintain cell migration and organ development (Treves et al., 2000; Mahmood et al., 2010; Hou et al., 2018). Patel et al. (2014) performed the whole-mount *in situ* hybridization of *Asph* at embryonic day 11.5 (E11.5) and E12.5 mouse embryos. They found a strong expression of *Asph* in snout, limbs, and eyes (Patel et al., 2014). To date, *ASPH* mutations identified in patients were established to be associated with Traboulsi syndrome which is characterized by facial dimorphism, lens dislocation, anterior segment abnormalities, and spontaneous filtering blebs (OMIM:601552) (Patel et al., 2014; Chandran et al., 2019), but does not refer to the abnormal bone mass. The knock-out *Asph* mice also replicated similar phenotypes with these patients (Dinchuk et al., 2002; Yuan et al., 2007; Patel et al., 2014). However, Peggy et al. demonstrated that *ASPH* expressed differentially in hBMSCs and was thought to be associated with the BMD and risk of bone fracture (Benisch et al., 2012). GWAS results suggested that a mutation occurred in 3’ region of *ASPH* might be related to bone mass (Koller et al., 2010). In addition, the previous study suggested that *ASPH* played great roles in the other key signaling pathways like Wnt signaling, Notch signaling and so on, which are all closely involved in bone formation (Tomimaru et al., 2013; Hou et al., 2018). As expected, our study found that *ASPH* was an important modulator during osteogenesis. The correlation analysis suggested that *ASPH* had positive correlation with osteogenic markers. The expression of *Asph* was also increasing during osteogenic differentiation. Moreover, the depletion of *Asph* inhibited osteogenic differentiation while the overexpression of *Asph* promoted osteogenic differentiation. Nevertheless, Mantila Roosa et al. (2011) suggested that *Asph* was downregulated during matrix formation, which is not consistent with our results. In this study, stable expression at common region of *ASPH* (exon 1–3) and decreased expression at exon 4–25 have been showed in Figure 3B, which indicated that junctin has increased during aging and senescence. Thus, we suspected that *ASPH* different isoforms might be involved in the same process with opposite role.

Further study revealed that the deficiency of *Asph* in BMSCs resulting in the dysfunction of osteogenesis and cellular senescence. Conversely, overexpression of longest *Asph* variant 1 in BMSCs restored the capacity of osteogenic differentiation and prevented the cellular senescence. Gsk3β has been reported to be regulated by *ASPH* inhibitor in hepatocellular carcinoma (Iwagami et al., 2016). In addition, Gsk3β can phosphorylates the sites of β-catenin at the sites of Thr41/Ser37/Ser33 followed by the degradation of β-catenin (Wu and Pan, 2010; Peng et al., 2020b). Gsk3β-mediated Wnt signaling can promote bone formation and prevent cellular senescence (Ye et al., 2007; Seo et al., 2008; Gillespie et al., 2013). Thus, we investigated if *ASPH* regulated osteogenic differentiation and cellular senescence in BMSCs through regulating Gsk3β or β-catenin. Of note, positive correlations of *ASPH* with *GSK3B* and *CTNNB1* have been found in the normal tissues, cancer tissues as well as cancer cell lines. Furthermore, we confirmed these positive correlations *in vitro*. The deficiency of *Asph* inhibited the accumulation of Gsk3β. Nevertheless, the non-phosphorylation of β-catenin at Thr41/Ser37/Ser33 has decreased which explained the downregulation of total β-catenin. The immunofluorescence analysis supported the downregulated Wnt signaling as well.

Of note, previous studies suggested that a relatively low level of *ASPH* expression in normal mature tissues, while abundant expression of *ASPH* in a variety of malignant tumors (Lavaissiere et al., 1996; Lin et al., 2019). Thus, *ASPH* has been thought to be a potential therapeutic target for different cancers (Shimoda et al., 2012; Dong et al., 2015; Sturla et al., 2016). However, according to our current data *in vitro*, it suggested that the treatment of *ASPH* inhibitor in patients with cancer need to be concerned because of their potential risks of bone loss or bone fracture.

## Data Availability Statement

Publicly available datasets were analyzed in this study. This data can be found here: the GEO database (Accession number: GSE35955; https://www.ncbi.nlm.nih.gov/geo/query/acc.cgi?acc~=~GSE35955).

## Ethics Statement

The studies involving human participants were reviewed and approved by the Ethics Committee of Xiangya Hospital of Central South University. The patients/participants provided their written informed consent to participate in this study. The animal study was reviewed and approved by the Xiangya Hospital of Central South University Ethics Committee.

## Author Contributions

HP designed this study, carried out most of the experiments, and wrote the manuscript. QG, TS, YX, T-JJ, and L-JG helped to collect samples. MW supervised the experiments, analyzed results, and proofread the manuscript. All authors contributed to the article and approved the submitted version.

## Conflict of Interest

The authors declare that the research was conducted in the absence of any commercial or financial relationships that could be construed as a potential conflict of interest.

## Footnotes

1 https://www.ncbi.nlm.nih.gov/geo/

2 http://cancergenome.nih.gov/

3 https://www.gtexportal.org/

4 https://portals.broadinstitute.org/ccle
